# Harnessing Artificial Intelligence in Health Research in Low-Income and Middle-Income Countries: Potential and Caution

**DOI:** 10.1016/j.mcpdig.2026.100380

**Published:** 2026-06-09

**Authors:** Siphamandla Bonga Gumede, Willem Daniel F. Venter, Nolwazi Mndebele, Samanta Tresha Lalla-Edward

**Affiliations:** Ezintsha, Faculty of Health Sciences, University of the Witwatersrand, Johannesburg, South Africa

The rapid expansion of artificial intelligence (AI) in health research has generated both considerable enthusiasm and important concerns. Although AI offers new opportunities to accelerate discovery, improve prediction, and optimize health system performance, its growing influence also raises critical questions about equity, validity, and real-world applicability, particularly in low- and middle-income countries (LMICs).^1^^,^^2^ In these settings, where structural constraints shape both data availability and healthcare delivery, the implications of adopting AI are especially consequential.^1^

In LMICs, where human resource constraints, data fragmentation, and infrastructural inefficiencies remain major barriers to health system performance, the potential utility of AI is considerable.^3^, ^4^, ^5^ Recent advances have reported its application in identifying individuals at elevated risk for HIV infection, supporting differentiated models of care for noncommunicable diseases, and analyzing patient-reported outcomes, signaling a shift toward data-driven and context-aware health interventions.^6^

However, the rapid proliferation of AI tools also raises substantive concerns. Many models are developed and validated in high-income settings, with limited consideration for the sociotechnical, epidemiological, and infrastructural realities of LMIC contexts. Challenges related to data quality, model transparency, interpretability, and algorithmic bias are well documented, and there remains a dearth of empirical evidence on the real-world effectiveness and equity implications of AI in health research.^7^ Furthermore, ethical dimensions, particularly regarding consent, data governance, community engagement, and power asymmetries, are insufficiently addressed in many AI-focused initiatives in global health.^7^

This commentary critically examines the promise and limitations of AI in health research, drawing on practical insights from work in HIV prevention and care, NCD management, clinical trials, and health system strengthening in LMICs. Framing the discussion through a pragmatic approach, we consider the role of implementation science in supporting the responsible integration of AI tools and technologies. Ultimately, we argue that while AI presents valuable opportunities for innovation, its success depends on careful alignment with ethical principles, contextual relevance, and the priorities of the populations it aims to serve. The AI applications discussed were purposively selected based on their relevance to health research, emerging use of AI in health research, and their ability to illustrate ethical and implementation challenges rather than to provide an exhaustive review.

In this commentary, we argue that while AI holds substantial promise for advancing health research in LMICs, its value will depend less on technological sophistication and more on how well it is aligned with context, equity, and implementation realities. Without deliberate attention to data representativeness, ethical governance, community engagement, and health system integration, AI risks reinforcing existing disparities rather than alleviating them.^1^^,^^2^ Conversely, when embedded within implementation science frameworks and co-developed with communities, AI can serve as a pragmatic tool to strengthen health systems and improve research relevance in resource-constrained settings.^8^, ^9^, ^10^ (Figure 1)^11^

**Figure 1:**
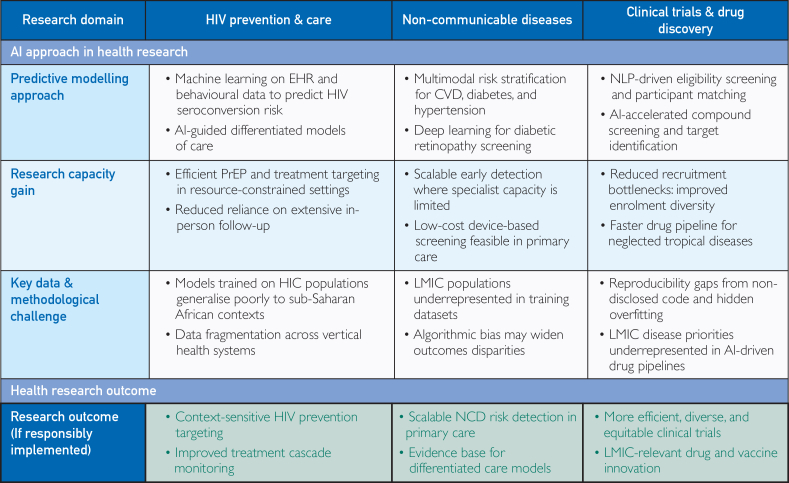
Illustrative examples of artificial intelligence applications deployed across health research domains in LMICs. Notes: These case studies or pilot projects leverage existing health data infrastructure and research networks to achieve greater scalability, higher methodological rigor, and improved research outcomes. Illustrative examples focus on health research applications rather than direct health care delivery. CVD, cardiovascular disease; EHR, electronic health records; HIC, high-income country; LMIC, low- and middle-income country; NCD, noncommunicable disease; NLP, natural language processing; PrEP, pre-exposure prophylaxis. Figure 2 from *Nature Health*.^11^

### The Promise of AI in Health Research

Artificial intelligence is demonstrating a transformative impact across numerous domains of health research by addressing historical challenges related to data complexity and resource constraints. In LMIC settings, where HIV incidence remains high and prevention resources must be carefully targeted, predictive models leveraging routinely collected clinic data, pharmacy records, and behavioral indicators have been used to identify individuals at elevated risk of HIV seroconversion.^12^ For example, risk prediction tools applied to programmatic data from public-sector HIV services have enabled more efficient targeting of pre-exposure prophylaxis and testing services in resource-constrained environments, where universal coverage is often not feasible.^6^^,^^9^^,^^12^

For instance, in ongoing work focused on AI-enabled self-care models in private pharmacy settings in South Africa, our project is exploring the integration of digital decision support tools to enhance client self-management. In this approach, individuals undergo digital screening and receive tailored guidance, followed by prompt linkage to pharmacy-based HIV services for pre-exposure prophylaxis initiation and continuation. These pathways are supported by interconnected digital systems that enable clinical decision support, real-time data capture, and follow-up tracking, allowing client journeys to be monitored from initial engagement through to service uptake and retention. This illustrates how AI-informed systems can support targeted outreach, streamline service navigation, and strengthen continuity of care in resource-constrained settings. However, it also highlights key implementation challenges, including ensuring data completeness, achieving interoperability across platforms, integrating with existing workflows in private pharmacy settings, and addressing privacy and data governance considerations in LMIC contexts.

Similarly, in managing chronic diseases such as cardiovascular disease, diabetes, and hypertension, AI algorithms integrate multimodal data to perform risk stratification. In many LMIC contexts, where specialist care is limited and health systems are overburdened, such tools can support task-shifting by enabling primary care providers to identify high-risk patients earlier and prioritize interventions within constrained resource settings.^8^^,^^13^

AI has also demonstrated substantial promise in medical imaging, where deep learning algorithms analyze radiological scans and pathology slides to detect conditions such as cancer and diabetic retinopathy.^14^ In LMICs, where access to specialists like radiologists and dermatologists is often limited, AI-assisted diagnostic tools have the potential to expand access to care and reduce diagnostic delays.^15^ For example, recent evidence from LMIC-focused studies suggests that AI-based dermatological diagnostic tools can achieve high accuracy while improving access to specialist-level assessment in resource-constrained settings.^15^

In the realm of clinical trials, AI tools employing natural language processing and machine learning have shown promise in automating eligibility screening, optimizing participant matching, and reducing recruitment bottlenecks. These approaches are particularly relevant in LMIC settings, where trial infrastructure may be fragmented and under resourced, and where identifying eligible participants efficiently remains a major operational challenge.^16^ By improving recruitment efficiency and data completeness, AI has the potential to enhance both the feasibility and representativeness of clinical research conducted in these settings.^17^, ^18^, ^19^

More recently, large language models are being explored as tools to support frontline health care and research activities in low-resource settings, with emerging evidence suggesting their potential to assist with clinical decision support, information synthesis, and task-shifting in contexts where human resources are limited^20^^,^^21^ (See Table).

**Table:** Key AI Applications in Health Research in LMICs: Opportunities and Cautions

Domain	AI application	Potential benefit in LMICs	Key caution
HIV prevention	Predictive modeling using EHR and behaviural data to identify seroconversion risk	Efficient targeting of prevention in resource-constrained settings	Models trained on HIC data may not generalize to LMIC populations
NCD management	Risk stratification for CVD, diabetes, and hypertension using multimodal data	Prioritizes early detection where specialist capacity is limited	Algorithmic bias may worsen outcomes for under-served groups
Medical imaging	Deep learning for cancer detection and diabetic retinopathy from radiology/pathology	Augments diagnostics in low-radiologist settings	Requires high-quality imaging infrastructure often absent in LMICs
Clinical trials	NLP and ML for eligibility screening, recruitment optimization, and adaptive trial design	Reduces bottlenecks and improves enrollment diversity	Reproducibility gaps undermine trust in AI-generated trial outputs
Drug discovery	Accelerated target identification, compound screening, and trial design	Reduces time and cost of therapeutics development	LMIC disease priorities may not align with AI-driven commercial drug pipelines
Remote monitoring	Real-time patient-reported outcomes and wearable signals for mental health and adherence	Enriches clinical datasets in dispersed populations	Digital divide limits access; and data privacy risks in low-regulation environments

Abbreviations: AI, artificial intelligence; CVD, cardiovascular disease; EHR, electronic health records; HIC, high-income country; LMIC, low- and middle-income country; ML, machine learning; NCD, noncommunicable disease; NLP, natural language processing.

### Challenges and Caveats

Despite its transformative potential, the integration of AI into health research presents critical challenges that threaten its validity and equity. These issues can be understood as a cascade of interrelated problems, beginning with the data itself and extending to the models, their real-world application, and their ethical governance.

Foremost among these challenges is the foundational issue of data quality and representativeness. Artificial intelligence models are entirely dependent on their input data, and when trained on incomplete, inconsistent, or biased datasets, particularly those derived from urban or tertiary care settings, they inevitably learn and amplify these biases.^22^^,^^23^ This problem is especially acute in LMICs, where structural disparities in data infrastructure mean that AI models often work less well for under-served populations, potentially deepening existing health inequities.^1^^,^^24^ The result is a portability gap that severely limits the generalizability of many AI systems developed in high-income countries.^22^^,^^25^ These limitations are particularly pronounced in this context, where data constraints and structural inequities directly shape model performance and downstream health outcomes.^2^

Compounding the problem of data bias is the epistemological challenge posed by the black box nature of complex AI models. Even if a model is trained on representative data, the complexity of deep learning architectures can make their decision-making processes difficult to interpret. This lack of explainability is not just a technical inconvenience; it directly undermines scientific methodologies. A model might achieve high predictive accuracy but fail to provide clear, interpretable insights into the underlying biological mechanisms, creating a correlation without causation dilemma that stifles etiological discovery.^26^ This lack of clarity also undermines clinical trust and impedes the integration of AI tools into care pathways.

Emerging approaches in explainable AI aim to address these challenges by improving the transparency and interpretability of model outputs, enabling clinicians and researchers to better understand how decisions are generated. In LMIC contexts, where trust and accountability are critical, integrating explainable AI approaches into AI-enabled systems may also support more meaningful community engagement by facilitating clearer communication of how data are used and how algorithmic decisions are made.^26^

Despite its transformative potential, AI health research is beset by a substantial reproducibility crisis. Many published models cannot be replicated because the original code, data, and training pipelines are not shared, and even when code is available, non-deterministic training processes and preprocessing variability can prevent independent replication.^27^ Moreover, models that appear to perform well on development datasets frequently fail to generalize to external cohorts because they have learned site-specific noise rather than underlying biological signals, a form of hidden overfitting that undermines scientific stability and external validity.^28^ These issues are further exacerbated by insufficient external validation and inadequate reporting methods, which collectively erode trust in AI findings and impede their translation into reliable clinical practice.

Beyond these scientific hurdles, the rapid evolution of AI in LMICs presents a formidable regulatory challenge. The current framework for software as a medical device is designed for static tools, not for dynamic AI models that continuously learn from new data. This mismatch creates a fundamental dilemma: how can regulators approve a model that will change after approval? Ensuring the ongoing safety, efficacy, and equity of these living algorithms requires new regulatory paradigms and continuous monitoring frameworks—largely still in their infancy.^29^

Ultimately, all these technical challenges are underpinned by ethical considerations that are frequently underexplored. The questions of who benefits from AI, who bears the risk, and who has control over the data are paramount. Key issues include the adequacy of informed consent for secondary data use, meaningful community engagement, data security, and the governance of data ownership and benefit sharing.^30^ Without explicit attention to these dimensions, AI innovations risk reinforcing, rather than alleviating, historical injustices and power asymmetries in global health research.

Beyond well-recognized concerns related to bias and data governance, the use of AI in health research may give rise to additional unintended consequences. Over-reliance on algorithmic outputs risks diminishing critical clinical and scientific judgment, particularly in settings where AI tools are perceived as authoritative or where human expertise is already overstretched. This may contribute to the deskilling of clinicians and researchers, as decision-making processes become increasingly mediated by opaque models.^31^ Closely related is the dilution of accountability, whereby responsibility for errors or harms is ambiguously attributed to algorithms rather than to the individuals or institutions deploying them.^32^ At a systems level, unequal access to AI infrastructure and expertise may further entrench institutional disparities, advantaging well-resourced research centers while marginalizing sites lacking technical capacity, thereby widening existing unintended inequities within and across health systems. These challenges are consistent with emerging qualitative evidence from LMIC settings, which highlights concerns related to feasibility, acceptability, and the integration of AI tools into routine care environments^33^ (Figure 2)^11^.

**Figure 2:**
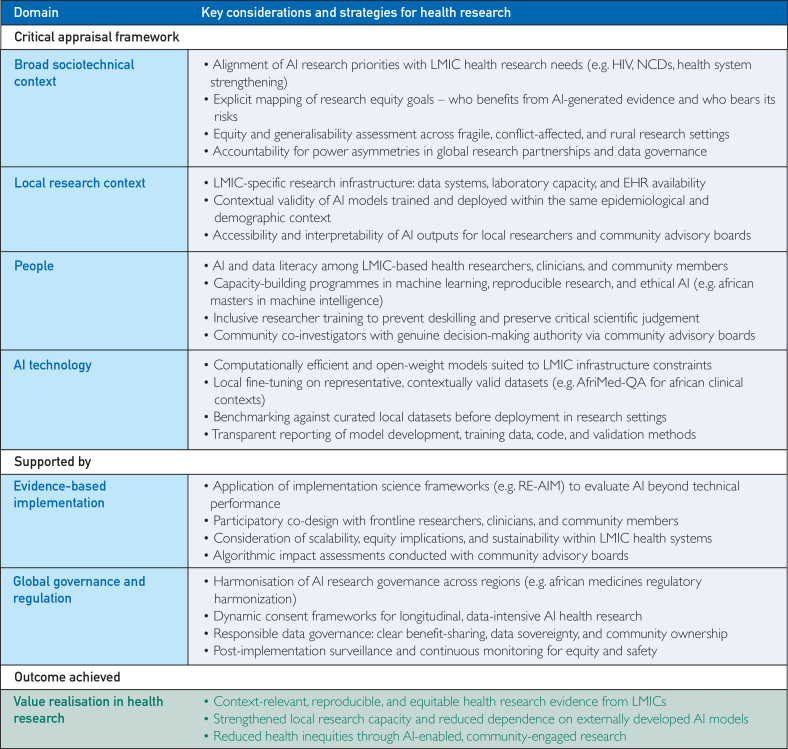
Key considerations and strategies for responsible artificial intelligence integration in health research in LMICs. Notes: The domains outlined within this critical appraisal framework represent overarching categories of implementation strategy for health research and are probably nonexhaustive. Systematic appraisal of these domains can inform the design of pragmatic studies grounded in LMIC contexts. EHR, electronic health records; LMIC, low- and middle-income country; NCD, noncommunicable disease; RE-AIM, Reach, Effectiveness, Adoption, Implementation, Maintenance. Table 2 from *Nature Health*.^11^

### Community Engagement for AI in Health Research

To counter these risks of perpetuating unintended consequences, the ethical imperative for community engagement is widely acknowledged, but a critical gap remains in translating this principle into practice. The question is not if we should engage communities, but how we can do so effectively. Several established frameworks can support the transition from tokenistic consultation to meaningful partnership.

A foundational approach is the adoption of Community-Based Participatory Research and co-production principles. These frameworks position community members not as subjects, but as co-investigators throughout the AI research lifecycle. A direct application is the establishment of a community advisory board with genuine decision-making authority, involved from problem definition to data governance and model validation. For this to be pragmatic, it must be properly resourced, with community partners compensated for their time and expertise. Such participatory approaches are well established and widely endorsed across public health, social science, and implementation research. However, their adoption in AI-related research and use remains limited, despite AI systems increasingly shaping decisions that directly affect communities.^34^

To guide the quality of engagement, Arnstein’s Ladder of Citizen Participation provides a useful framework for assessing whether engagement reflects consultation or genuine power sharing.^35^ The ladder ranges from non-participation to consultation, placation, and ultimately partnership, delegated power, and citizen control.^35^ Setting a goal to reach levels such as partnership or delegated power offers a clear trajectory for deepening engagement in AI and health research.^35^

A more specific tool tailored to the risks of AI is the algorithmic impact assessment. a structured process for proactively assessing the potential benefits, biases, and social harms of an algorithmic system; it has been proposed as an accountability mechanism that foregrounds impacts rather than just technical performance.^36^ When conducted in collaboration with a community advisory board, algorithmic impact assessment can move beyond technical audits to include perspectives from those most affected, helping surface context-specific adverse impacts and co-develop mitigation strategies.^37^

Finally, the principle of respect for persons demands rethinking consent for long-term, data-intensive AI research. Dynamic or ongoing consent options offer a pragmatic solution.^38^^,^^39^ Unlike one-time broad consent, dynamic consent uses digital platforms to maintain an ongoing relationship with participants, allowing them to choose how their data is used in future studies and to withdraw consent for specific purposes. Empirical research shows that dynamic consent enhances participant control, transparency, and trust, enabling real-time updates, personalized preferences, and greater autonomy in decision-making.^38^^,^^39^ These approaches can enhance transparency, strengthen trust, and support participant autonomy in evolving research contexts.

However, implementing such approaches in LMIC settings may be constrained by varying levels of digital literacy, limited access to technology, and language or literacy barriers, particularly in rural populations. These factors may affect individuals’ ability to fully understand and engage with consent processes, underscoring the need for contextually appropriate, accessible, and culturally sensitive consent strategies.

Deepening community engagement in AI health research is achievable by adopting these established yet adaptable frameworks. For researchers, the path forward is clear: integrate these pragmatic approaches, share power, and resource partnerships adequately. By doing so, we can ensure that the AI revolution and innovation in health are guided by the needs of the population they intend to serve.

### Ethics, Equity, and Implementation Science

Ethical AI in health research extends beyond technical considerations of bias and transparency to encompass questions of fairness, justice, and accountability in real-world implementation. In LMICs, where historical inequities shape both data generation and health system capacity, the extraction of data from marginalized communities without meaningful benefit sharing risks reinforcing existing power asymmetries and perpetuating inequitable global research dynamics in practice, not just theory.^40^

Implementation science offers a pragmatic framework for addressing these challenges by shifting attention from whether AI tools work to how, for whom, and under what conditions they deliver value. Frameworks such as Reach, Effectiveness, Adoption, Implementation, and Maintenance enable systematic evaluation of AI interventions beyond performance metrics, incorporating equity, sustainability, and contextual fit as core outcomes.^41^

Applying an implementation science lens is particularly important for AI-enabled interventions in disease prevention and management, where social determinants of health, health system constraints, and workforce capacity strongly influence effectiveness. Participatory co-design processes involving clinicians, patients, and frontline implementers can further enhance the relevance, acceptability, and trustworthiness of AI tools, while clarifying accountability and supporting responsible scale-up.

### Looking Ahead: Responsible Integration

To unlock AI’s full potential in health research, several priorities must be addressed. Investment in local technical capacity and infrastructure is essential to reduce dependence on externally developed models and ensure sustainability. Equally important is the development of context-aware algorithms trained on diverse and representative datasets. The inclusion of ethicists, community representatives, and social scientists in the design, governance, and evaluation of AI projects can help ensure these tools align with ethical standards and community priorities. A strong commitment to open science and transparency in model development and reporting will be critical to fostering trust, accountability, and replicability.

Recent work has further highlighted the importance of equity-focused frameworks for emerging AI technologies, including large language models, emphasizing the need for contextually appropriate design, inclusive data practices, and governance mechanisms that prioritize equitable adoption in LMIC settings.^42^

However, the financial implications of implementing AI in health research and service delivery in LMICs warrant careful consideration. The development, deployment, and ongoing maintenance of AI systems require substantial investment in infrastructure, data systems, workforce training, and continuous model updating. In resource-constrained settings, these costs may place additional strain on already overburdened health systems. At the same time, there is growing recognition that, when appropriately implemented, AI has the potential to improve efficiency, optimize resource allocation, and reduce long-term costs by supporting targeted interventions, reducing unnecessary service utilization, and enhancing retention in care. As such, incorporating cost-effectiveness considerations into the design and evaluation of AI-enabled interventions will be critical to ensuring their sustainability and scalability in LMIC contexts.^8^

## Conclusion

Artificial intelligence offers considerable potential to advance health research in LMICs, particularly in domains such as HIV prevention, NCD management, and health systems strengthening. However, the extent of its impact will be determined not solely by technical innovation but by the degree to which its development and deployment are informed by empirical evidence, contextual relevance, and ethical integrity. Researchers must guard against technological determinism and approach AI as a complementary tool, one that must be rigorously evaluated, equitably implemented, and transparently governed. Only through such a multidisciplinary, community-engaged, and ethically grounded approach can AI meaningfully contribute to the advancement of health research.

## Potential Competing Interests

The authors report no competing interests.

## Declaration of Generative AI and AI-Assisted Technologies in the Writing Process

During the preparation of this work the authors used Perplexity AI and ChatGPT to assist in reviewing the clarity, grammar and coherence of the manuscript content during development. After using this tool, the authors reviewed and edited the content as needed and takes full responsibility for the content of the publication.
